# Alteration in the potential of sediment phosphorus release along series of rubber dams in a typical urban landscape river

**DOI:** 10.1038/s41598-020-59493-3

**Published:** 2020-02-17

**Authors:** Linlin Bao, Xuyong Li, Jingjun Su

**Affiliations:** 1 State Key Laboratory of Urban and Regional Ecology, Research Center for Eco-Environmental Sciences, Chinese Academy of Sciences, Beijing, 100085 China; 2 College of Resources and Environment, University of Chinese Academy of Sciences, Beijing, 100049 China

**Keywords:** Environmental monitoring, Element cycles

## Abstract

Rubber dams are widely used for landscaping in urban rivers and they retain large amounts of sediments. The sediments are rich in phosphorus (P) which can cause river eutrophication. Little is known about P release in rubber dams. We investigated the potential of sediment P release by isotherm experiment in an urban river with 30 rubber dams of northern China. We found that the potential of sediment P release (percentage saturation of zero equilibrium P concentration, EPC_sat_) was 76% at natural river part above dams, and then decreased to 67% at the 4^th^ dam because of high deposition of fine sediments within the upper 4 dams. Between the 5^th^ and the 30^th^ dams, EPC_sat_ increased to 90% because of the decrease of fine sediments and water soluble reactive P. EPC_sat_ was also significantly higher (*p* < 0.05) in April and August than in November. The results suggest that the potential of sediment P release in this dammed river was mainly controlled by sediment grain size and biological effects. Therefore, management strategies for dammed rivers should focus on reducing P inputs and improving the hydraulic conditions.

## Introduction

Phosphorus (P), an essential nutrient for primary productivity, is generally the limiting element in freshwater systems. Many rivers worldwide have degraded water quality and frequently become eutrophic because of excessive P inputs and weak hydrodynamic forces by dams^1,2^. As dam construction usually results in more P retention in the sediments within impoundments^3,4^, sediment P, therefore, becomes one of the main causes for eutrophication of dammed rivers^5,6^. Dams, ranging from large, well-established, reservoir dams^6–8^, to small dams, including weirs and rubber dams, can all efficiently retain sediments that contain P^8–10^. Rubber dams are widely constructed in urban rivers, especially in China, for water storage and landscaping. Several study have found that the potential of P release was higher from sediments retained by rubber dams than from sediments downstream of the dams^10^, which could contribute to eutrophication of the impounded river^9^. However, less attention has paid to the role of small dams in urban rivers in P transport between water and sediments. To help mitigate eutrophication and improve the health of aquatic ecosystems, we need more information about the potential of P release from sediments retained behind dams in urban rivers.

It has been report that P is more likely to be released from lake sediments than river sediments because of differences in hydraulic conditions, vegetation communities, and other physicochemical factors^11–14^. Generally, the potential of sediment P release is controlled by the physicochemical properties of both the sediments and the water column, which were mainly studied in lakes^15–17^. On one hand, the potential of sediment P release depends on the contents of total P (TP) and the P fractions, and other physicochemical components of the sediments. Studies have shown that, as the concentrations of TP and, particularly, in which bioavailable P (BAP) increase, the potential of sediment P release generally increases^18,19^. The Standards, Measurements and Testing Program of the European Commission (SMT) method^20^ defines a range of sediment P fractions that might be involved in release processes, including exchangeable labile P (ex-P), apatite P (Ca-P, calcium associated forms), non-apatite P (Fe/Al-P, the forms associated with oxides and hydroxides of Al, Fe, Mg and Mn), and organic P (OP); of these, ex-P, Fe/Al-P, and OP are considered as BAP and can easily release or be mineralized to water for maintaining its eutrophic state over a long term^6^. Other physicochemical components of the sediments, including the contents of fine particles^21^, organic matters (OM)^22^ and hydratable metals, like Al and Fe^23^, all have negative relationship with sediment P release. On the other hand, P concentration in the water body can also directly influence the state of sediment P release. For example, when water dissolved P concentration is lower than the zero equilibrium P concentration (EPC_0_, the critical phosphate concentration in contact with the sediments that causes no net release or uptake of P)^18,24^, P will be released from sediments. Therefore, when external P sources were controlled, P release from sediments would increase^25–27^. Other properties of the water column, such as pH^21,28^, oxygen status^29,30^, temperature^9,31^ and flow conditions^32,33^, may all contribute to P release from sediments into the water column. To date, few studies have fully and systematically considered how these physicochemical factors of sediments and water column vary when small dams are constructed in river systems.

Previous researches have demonstrated that there are differences of physicochemical properties of sediments and water column between reservoirs and free-flowing river systems. For instance, studies have shown that there was more P in sediments near reservoir dam than in sediments of the upper river^34,35^, and sediment BAP increased from the reservoir inlet to the reservoir dam^36^. It was also reported that sediments had higher contents of clay (finer grain size)^37^ and OM^38,39^ in dammed rivers than in natural rivers. Furthermore, reservoir dams generally cause a decrease in the flow velocity by increasing the water residence time; and the P concentration in the water column would decrease when particles settle and P is assimilated within reservoirs^40,41^. These variations in sediments and water column of rivers with dams may have implications for the potential of P release and then cause long-term eutrophication of the impoundments. Therefore, it is practical to study how these variations in physicochemical factors of dammed rivers would affect the variations in potential of sediment P release.

In cascade reservoirs along Lancang River, longitudinal variability of phosphorus species and its relation metals and grain size distribution of sediments were well studied but no further information about their correlations with the potential of sediment P release was offered^36,42^. In this study, we investigated the potential of sediment P release in a typical urban landscape river with 30 rubber dams that flowed through the center of Zhangjiakou city, Hebei Province, northern China. Our previous study about the typical urban landscape river with 30 rubber dams has revealed that large amounts of sediments and P were retained behind the dams^43^. Therefore, in order to further elucidate the effects of dams on sediment P release, the following questions will be addressed in present study: (1) if the potential of sediment P release varied with distance downstream along the series of rubber dams, (2) which physicochemical factors, both from the sediments and the river water, primarily controlled the potential of sediment P release in this dammed river, and (3) whether the presence of dams, by interfering with the physicochemical environment, affected the potential of sediment P release in this river.

## Sampling and Analysis Methods

### Sample collection

**Figure 1:**
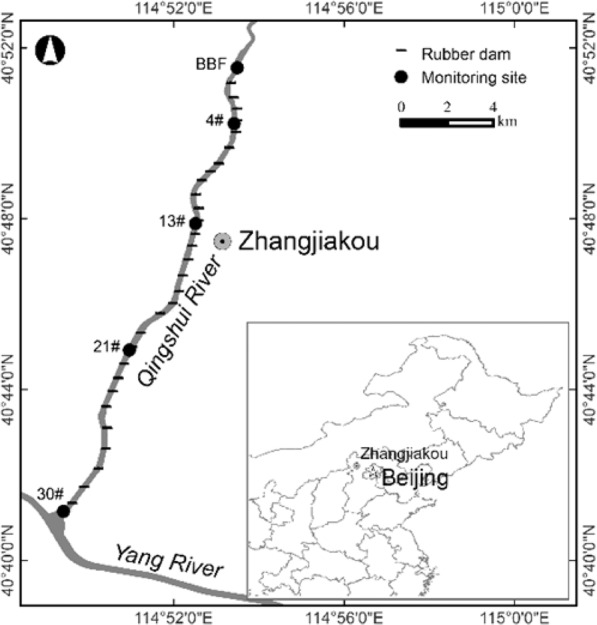
Map of the dammed urban landscape river and sampling location.

Study area and sampling sites are showed in Fig. 1. The urban landscape river, which is the main channel of the Qingshui River, is 22 km long and, on average, 120 m wide. Along the river, there are 30 tap-water-filled rubber dams, numbered in this study as 1# to 30#. The river channel is totally impermeable and has a reinforced-concrete riverbed and vertical banks, and, in principle, only water from the upper watershed discharges into this urban river. Based on our previous study^40^, sediment and water samples were also collected from Beibengfang (BBF) in the natural part of the river above the dams and from the impoundments of dams 4# (at which the Zhangjiakou Hydrological Station locates), 13#, 21#, and 30# in spring (April), summer (August), and autumn (November) of 2016. Sediment samples were collected in the upper 10 cm layer. At each site, at least three sub-samples were collected to mix into one sample. Altogether, we collected 15 sediment samples. The sediment samples were freeze-dried, homogenized, and passed through a 100-mesh (0.149 mm) sieve. Various physicochemical properties of the river water were measured *in situ* at the sampling sites, as follows. Velocity and flow were measured with a portable flow meter (Flowatch, Switzerland). Water temperature, oxidation-reduction potential (ORP), and pH were measured using an Ultrameter (6 Psi, Myron L Company). Dissolved oxygen (DO) was monitored using a portable LDO sensor (HQ40d, Hach Lange). The various P species in water samples were determined by the molybdenum blue method, including total P (W-TP), total dissolved P (W-TDP), soluble reactive P (W-SRP), and total particulate P (W-TPP, the difference between TP and TDP). The suspended sediments (SS) concentration was tested as turbidity units (NTU).

### Phosphorus absorption isotherm experiments and release potential

Laboratory equilibrium experiments were conducted to determine the zero equilibrium P concentration (EPC_0_, defined as the critical concentration below which P would be released from sediments to the water) of the collected sediments. Sediments (2.5 g) were placed in 50 mL centrifuge tubes with screw caps and were mixed with 25 mL of KH_2_PO_4_ equilibrating solution at a range of concentrations (0, 0.01, 0.025, 0.05, 0.1, 0.25, 0.5, 1, 2, 5, 8, 10, 15, and 20 mg-P/L). This set of initial high dissolved P concentrations allowed adsorption sites of sediments to reach saturation. It also covered the whole range of the prevailing P concentrations in the natural waters from various sources^24^. The mixtures were shaken at 150 rpm for 24 h, centrifuged, and then the supernatants were collected. Concentrations of soluble reactive P (SRP) in the supernatants were determined using the molybdenum blue method. All the samples were analyzed in duplicate and the mean SRP concentration were used. The sorption processes were represented with a modified Langmuir isotherm model by Zhou *et al*.^44^:

1 $$\Delta {\rm{Q}}=\frac{{Q}_{max}\cdot k{C}_{SRP}}{1+k{C}_{SRP}}-NAP$$

where Q_max_ (mg/kg) is the maximum P adsorption capacity when saturated, *C*_*SRP*_ (mg/L) is the initial SRP concentration of the equilibrating solution, *k* is the bonding energy constant (L/mg). NAP (mg/kg) is the native adsorbed exchangeable P, and ∆Q (mg/kg) is the P content adsorbed by the sediments. The *x* intercept of the curve is the EPC_0_ (mg/L). Then An EPC_0_ percentage saturation term, EPC_sat_ (%) (Eq. 2), was calculated to determine the potential of sediment P release^45^:

2 $$EP{C}_{sat}=100\cdot \frac{EP{C}_{0}-{C}_{SRP}}{EP{C}_{0}}$$

When EPC_sat_ >0, there is the potential for P being released from the sediments to the river water. When EPC_sat_ <0, P will be adsorbed by sediments. An EPC_sat_ of zero represents equilibrium.

### Measurement of sediment properties

Contents of sediment P fractions were determined using a modified extraction method based on the Standards, Measurements and Testing Programme (SMT) method^46^. Details of the procedures are presented in Table S1.

Grain sizes of the sediments were determined as described previously^33^ with a laser particle size analyzer (Malvern master 2000). According to previous classification criteria^47,48^, the particle size fractions were separated into clay (<4 μm), silt (4–63 μm) and sand (>63 μm), representing fine sediments, medium-size sediments and coarse sediments respectively. The contents of major ions (Fe, Al, Ca, and Mg) that were related to P adsorption in the sediments were determined by ICP-OES^39^. Organic matters (OM) content was estimated as loss on ignition at 550 °C^46^. To check the accuracy of the analysis procedures, we tested a Chinese geochemical standard reference sample of lake sediments, GSS 9, at the same time.

### Statistical analysis

Differences between the different seasons of all the parameters were analyzed using one-way variance analysis (ANOVA) with independent samples t-test or non-parametric statistical methods with the Mann-Whitney U test in SPSS 16.0. The major influencial factors controlling the potential of sediment P release and their relationships were determined by principal component analysis (PCA). Then relative weight method^49^ in R language version 3.5.1 was used to determine the relative importance or contribution of various predictor variables to EPC_sat_. The figures were produced in OriginPro 8.0.

## Results

### Variations in the potential of sediment P release along the dammed urban river

**Figure 2:**
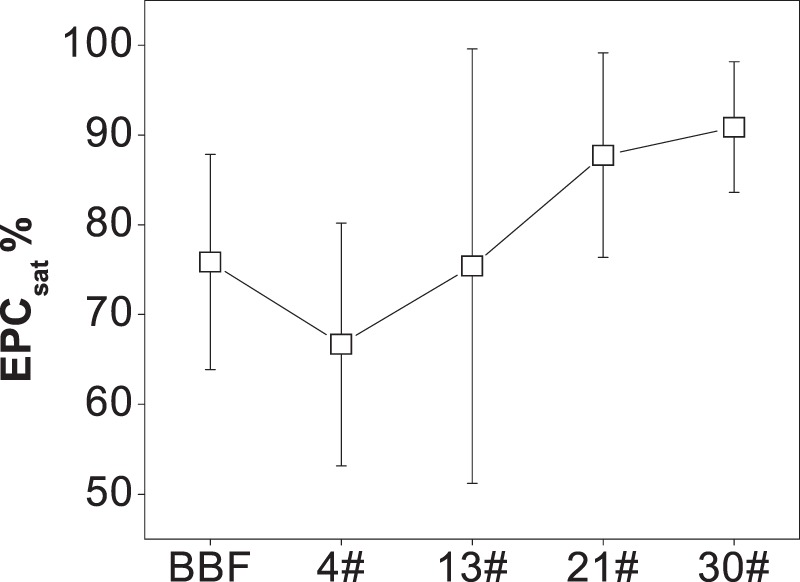
Variations in EPC_sat_ along the dammed river. Vertical bars indicate the standard errors of the seasonal average.

EPC_0_ and other parameters obtained from Eq. 1 were listed in Table S2. As shown by the *r*^2^ values, all of which were greater than 0.9, the model worked very well. EPC_sat_, calculated from Eq. 2, ranged from 38% to 100% (Fig. 2). The mean EPC_sat_ was 76% at site BBF, 67% at site 4#, and then increased until it reached 90% at site 30#. The values of EPC_sat_ were all greater than zero, which revealed that P would be released from the sediments in this river, and the potential of sediment P release was greatest at site 30#.

### Phosphorus forms in the sediments and water along the dammed river

**Figure 3:**
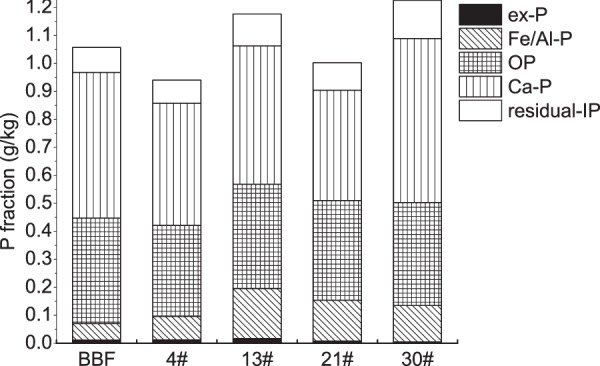
Average contents of the different P fractions (g/kg) in the sediments along the dammed river.

Contents of TP and P fractions in the sediments varied differently along the dammed river (Fig. 3). Sediment TP contents ranged from 0.79 to 1.32 g/kg, which was lower at sites 4# and 21# than at sites BBF, 13#, and 30#. The Ca-P, OP, Fe/Al-P, and ex-P contents ranged from 0.347 to 0.588 g/kg, 0.199 to 0.562 g/kg, 0.042 to 0.213 g/kg, and from 0.003 to 0.032 g/kg, respectively. Ca-P therefore dominated the P fractions in the sediments. Fe/Al-P and OP were the main fractions of BAP and determined the spatial variations in BAP. Except the sediment TP and BAP at the 4^th^ dam were the lowest, BAP contents in the sediments were also slightly lower at BBF than that within dams.

In the river water, the concentrations of W-TP, W-TPP, W-TDP and W-SRP were all higher at site BBF than at the lower sites (Fig. S1). Our previous study^40^ has already revealed that the majority of the sediments and P transported to the urban area were retained in the section of the river from site BBF to site 4# by sedimentation and biological effects, including uptake and assimilation. And due to biological P uptake and transformations, W-DOP concentrations increased slightly from site BBF to site 30#, while W-SRP decreased at the same time.

### Variations in other physicochemical properties of sediments and water along the dammed urban river

**Figure 4:**
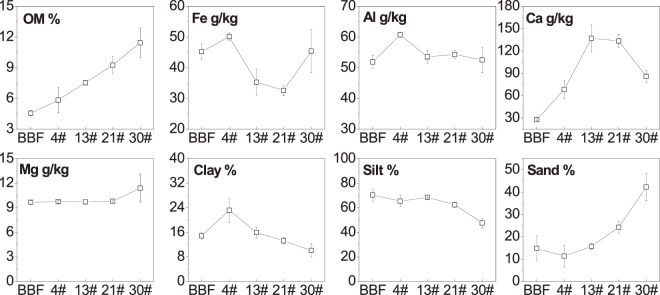
Chemical and physical compositions of the sediments. Vertical bars indicate the standard errors of the seasonal average.

Contents of OM, hydratable metals and the sediment grain size distribution are shown in Fig. 4. Sediment OM increased linearly from BBF to 30#, reflecting the effects of increase growth of macrophyte (*Potamogeton crispus*) and phytoplankton along the river channel^39,43^. Hydratable metals in the sediments were dominated by Ca (average concentration of 91 g/kg), Al (55 g/kg) and Fe (42 g/kg), and the Mg (10 g/kg) concentrations were relatively low. And sediment Ca were higher in the dammed areas than at site BBF. Sediment Fe contents were lower at sites 13# and 21# than at sites BBF, 4# and 30#. Sediment Al contents were higher at site 4# than at other sites. Sediment Mg contents were higher at site 30# than at other sites. The grain size distribution showed that silt was the main particles in the dammed urban river, with the percentage to the total particles decreasing from76% at site BBF to 53% at site 30#. While the percentage of sand varied oppositely, with 14% at site BBF and 42% at site 30#. Clay accounted for 15% at site BBF of the sediment particles, increased to 23% at site 4#, and then decreased to 10% at site 30#.

**Figure 5:**
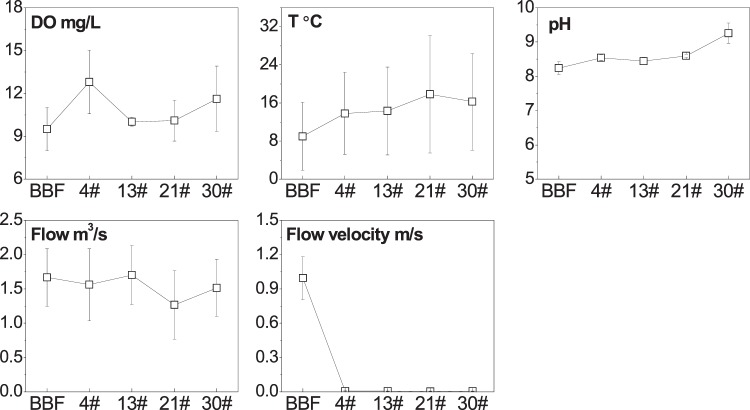
Variations in physicochemical properties in the water along the dammed river. The vertical bars indicate the standard errors of the seasonal average.

In the river water, DO ranged from 7 to 15 mg/L, with higher values at site 4# (Fig. 5). Water temperature (T) increased with distance downstream, which may reflect the fact that the surface area of open water, and therefore the solar radiation absorbed, was greater in the lower part of the river than in the upper part. The pH values increased and became more alkaline from BBF to 30#, probably because of photosynthesis by aquatic organisms within the river impoundments^15^. River flow was relatively continuous in this river, but the mean velocity deceased from 1 m/s at BBF to less than 0.01 m/s in the dammed sections, where the channel was widened when the dams were constructed.

### Seasonal variations in the potential of sediment P release and its influencing physicochemical factors

**Figure 6:**
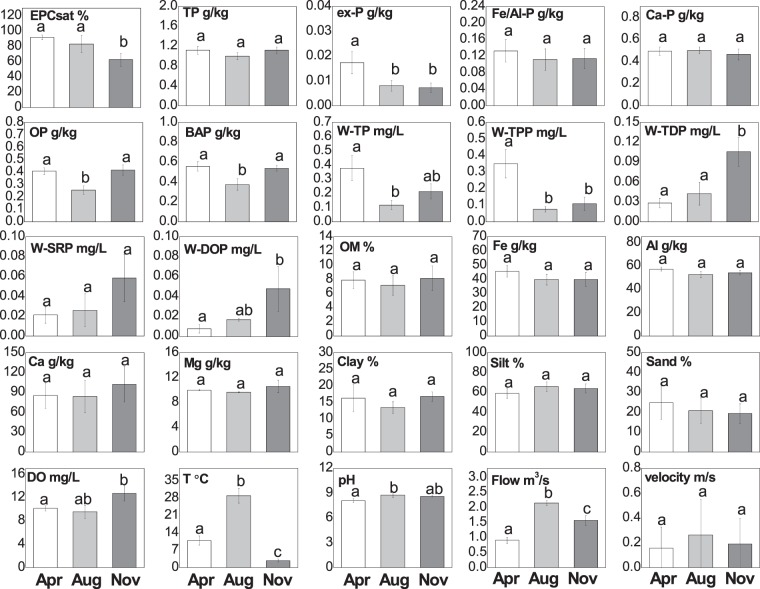
Seasonal variations in EPC_sat_ and physicochemical factors of the sediments and river water. The different letters (a, b and c) above each bar represent significant differences between seasons at *p* < 0.05.

EPC_sat_ values were significantly (*p* < 0.05) lower in November than in April and August (Fig. 6). The contents of TP, Fe/Al-P and Ca/P did not vary significantly in the three seasons, but sediment OP and BAP were significantly lower in August than in the other two seasons. And sediment ex-P were higher in April. No significant seasonal differences were found in the contents of sediment OM, hydratable metals and the grain size distribution. Concentrations of W-TP (mainly as W-TPP) were significantly higher in April than in the other two seasons, and W-TDP was higher in November, according with the variation in W-DOP. There were obvious seasonal variations in the water temperature, which was highest in August, followed by April, and was lowest in November. River flow was significantly higher in August than in November, reflecting the summer flood season, and was lowest in April. While flow velocity did not vary significantly between seasons.

### Contribution of physicochemical factors determining the potential of sediment P release in the dammed urban river

**Figure 7:**
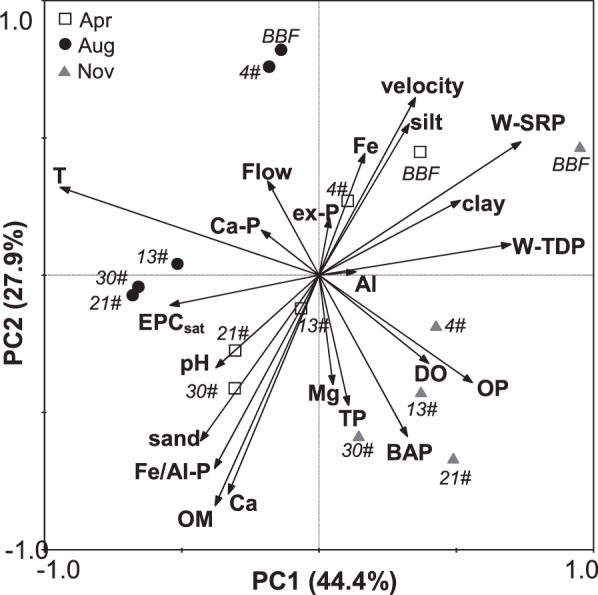
PCA ordination graph of EPC_sat_ and its influential physicochemical factors.

**Figure 8:**
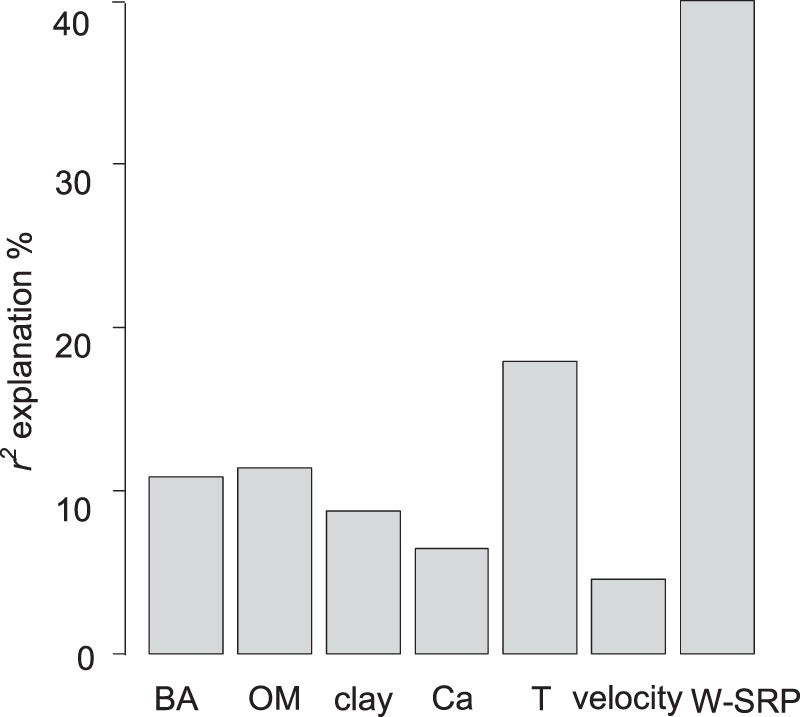
Contributions of the main physicochemical factors to EPC_sat_ variations (*r*^2^ = 0.73).

We evaluated the physicochemical factors of the sediments and water that had influences on the EPC_sat_ by PCA (Fig. 7). The first 2 principal components (PC1 and PC2) explained 44.4% and 27.9% of the variance respectively. Eleven factors, namely BAP, OM, silt, sand, Ca, water temperature, velocity, W-TDP, W-SRP, OP, and Fe/Al-P, that had correlation coefficients either greater than 0.5 or less than −0.5 with PC1 and PC2 were selected as the major controls on the EPC_sat_. To express the effects of these variables more clearly, we removed the collinear factors OP and Fe/Al-P (both included in BAP), and W-TDP (related to W-SRP), and replaced silt and sand with clay. Then the contributions or relative importance of the remaining variables to the EPC_sat_ were examined by the relative weight method (Fig. 8). It showed that W-SRP, which was negatively correlated with the EPC_sat_, contributed the most to the variation in the EPC_sat_ and explained 40.0% of the *r*^2^. Water temperature, sediment OM, BAP, and clay contributed 17.9%, 11.4%, 10.8%, 8.8% respectively for the variations. Water temperature and sediment OM were positively correlated with EPC_sat_, and BAP and clay were negatively correlated to the EPC_sat_. Sediment Ca (6.5%) and water flow velocity (4.6%) explained the least of the *r*^2^ and were positively and negatively correlated with the EPC_sat_, respectively.

## Discussion

In our study area, the hydraulic conditions have been changed drastically by the series of rubber dams, as flow velocity has dramatically decreased, especially between BBF and 4# (Fig. 5), and most of the SS were retained in the section before 4^th^ dam (Fig. S1). It resulted in a higher proportion of fine particles (clay) in sediments at site 4# than at BBF (Fig. 4). General trend of sediment grain size distribution along rivers shows that the sediments would gradually become finer in a downstream direction, because fine particles could travel longer distances than coarse particles^42,50^. However, the proportion of large particles (sand) then increased from site 4# to site 30#, perhaps because few fine particles were able to travel to the lower dams through SS, and coarse materials eroded from the artificial channel and deposited to the bed sediments. PCA analysis showed in our study that sediment clay was negatively correlated, and sand was positively correlated, with EPC_sat_, as P can be released more easily from the coarse particles than from the fine particles^21,51^. The variation in the flow velocity was negatively related to EPC_sat_, which is opposite to the results of previous studies that P could be adsorbed to sediments during periods of low flow but would be released or flushed away during high flows^32,33^. It was most probably because the impoundments within rubber dams offered optimal conditions for the growth of aquatic plants and algae^2^, thereby enhancing P release from sediments, as suggested by the decrease in W-SRP with distance downstream (Fig. S1).

Since the dammed urban river has suffered from bloom of macrophyte and phytoplankton alternatively every year, and the biomass of which usually increases with distance downstream, the variations in W-SRP, sediment BAP and OM might be also highly associated with the biological processes. In general, P concentrations in the river water within dams were low because of P deposition and adsorption to sediments and uptake by organisms^6,40^, with the dissolved P mainly taken up by organisms^52^. In this river, the growth of macrophyte or phytoplankton has caused the concentrations of W-SRP decreasing in a downstream direction^43^; there may be more P release from the sediments to support ongoing organism growth^26,53^. W-SRP concentrations were therefore negatively correlated with the EPC_sat_ (Fig. 7). The decomposition of OM can trigger P release from sediments to water^15,27,54^. Higher contents of sediment BAP are also associated with a higher potential of P release from sediments^19,36^. In this study, the contents of BAP were higher in the sediments retained behind downstream dams than at BBF. And BAP, especially for Fe/Al-P in which, was positively correlated with sediment EPC_sat_ (Fig. 7). We infer that higher biological activities within dams has caused higher active P exchange between water and sediments, resulting in more P assimilating and depositing by organisms to sediments. Moreover, the pH in river water increased from BBF to 30# (Fig. 5), which might have also been caused by the increase in photosynthesis because of the increase growth of macrophyte and phytoplankton^15^. Increasing pH could enhance the release of P from sediments, because, in alkaline conditions, the isoelectric points of the surfaces of sediment hydratable metals (e.g. iron(III) oxides) are largely negative meaning that P, as a negatively-charged ion, cannot interact with them or be adsorbed to sediments^55^. In summary, when the dams were constructed, the hydraulic conditions changed (the velocity decreased and the water retention time increased), which promoted the macrophyte and phytoplankton growth and altered the physicochemical properties of both the sediments and river water. It then affected the potential of sediment P release.

The water temperature, indicating the seasonal variation in weather and biological processes, was positively correlated with the EPC_sat_, as more P could be released from sediments in dams in warmer seasons^9^. Furthermore, the contents of BAP were significantly lower in August than in the other seasons (Fig. 6). It is because higher biological activities in summer period has caused more P releasing from the sediments. Seasonal variations in the water temperature, W-TDP and sediment BAP (mainly as OP) were all consistent with the seasonal variation in the EPC_sat_ in this dammed river. Therefore, combining the variations in hydraulic conditions, sediment size distribution and other physicochemical factors, we assumed that EPC_sat_ was lower at 4# than at BBF mainly because the sediments were finer at 4# than at BBF, while the increase of EPC_sat_ from 4# to 30# was due to the increase growth of organisms and the increase proportion of sediment sand along the series of dams.

In addition, dam construction had the same effects on the accumulation of both sediments and metals^39^. The variations in the proportion of sand in the sediments highlighted the role of the artificial channel as another pollution source. The high Ca contents within the dams might also reflect the pollutants release from the concrete materials^56^. There was positive relationship between Ca and EPC_sat_ in this study. It contrasts with the usual knowing that sediments can absorb more P when it is rich in hydratable metals (e.g. Fe, Al and Ca)^57^. We need to examine this further to clarify whether the absence of Ca-related interactions in this study is related to the presence of different Ca fractions that do not readily react with P. The fact that the TP contents in sediments fluctuated with distance downstream is also not consistent with our expectations as the sediment TP should be lower in the downstream than in the upper area due to cascading retention^34,35^. We assumed the only P input was the upstream river, however the lack of pattern in the contents suggests that there were additional external sources of pollution. Therefore, it is necessary to identify and control the specific pollution sources in this dammed urban river, in order to curb sediment P release and improve water quality.

## Conclusion

In this study, we examined how the potential of sediment P release, measured by the EPC_sat_, and physicochemical factors in the sediments and the river water, varied along an urban river with series of rubber dams. The results showed that the EPC_sat_ in the dammed river was 76% at a site above the dams (BBF), 67% at the 4^th^ dam, but increased to 90% at 30^th^ dam. Various physicochemical factors in sediments and water influenced the potential of sediment P release in this dammed river. We found that W-SRP, water temperature, and sediment OM and BAP explained most of the variation in the EPC_sat_, while the sediment grain size, Ca content, and the flow velocity were less influential. The growth of macrophyte and phytoplankton within the dams affected the variations in W-SRP concentrations and the sediment BAP and OM, which then influenced the EPC_sat_, indicating the indirect effects by biological effects of dam construction on the potential of sediment P release. Therefore, EPC_sat_ was higher in April and August than in November because of the water temperature as well as the corresponding biological activities. While the series of rubber dams has changed the flow velocity and the distribution of sediment particles that influenced EPC_sat_, reflecting a more direct way of dams to influence the potential of sediment P release. The results implied that the variations in potential of sediment P release in this dammed river was mainly driven by biological effects and sediment grain size. To control the release of P from sediments and eutrophication in similar urban rivers with dams, P inputs should be reduced and the hydraulic conditions in the dams should be optimized.

## Supplementary information

Supplementary information.
