# Supplementary material for: Alteration in the potential of sediment phosphorus release along series of rubber dams in a typical urban landscape river
Source: Sci Rep. 2020 Feb 17;10:2714. doi: 10.1038/s41598-020-59493-3 (PMC7026097; doi:10.1038/s41598-020-59493-3)
Supplement: Supplementary file 1 — Supplementary information. [file 41598_2020_59493_MOESM1_ESM.docx]

***Supporting information for***

**Alteration in the potential of sediment phosphorus release along series of rubber dams in a typical urban landscape river**

Linlin Bao^a,b^, Xuyong Li^a,b,^^[[1]](#footnote-1)^, Jingjun Su^a,b^

*a State Key Laboratory of Urban and Regional Ecology, Research Center for Eco-Environmental Sciences, Chinese Academy of Sciences, Beijing 100085, China*

*b College of Resources and Environment, University of Chinese Academy of Sciences, Beijing 100049, China*

**Table S1** P fractions and the extraction protocol^1-3^

| P fractions | Extraction method |
| --- | --- |
| ex-P | 0.2 g sediments with 20 mL of 1 M MgCl_2_ for 2 h shaking. |
| Fe/Al-P | Residue of ex-P, 20 mL NaOH 1M for 16 h shaking; and then 10 mL extract and 4 mL 3.5 M HCl for 16 h shaking. |
| Ca-P | Residue of Fe/Al-P, 20 mL HCl 1 M for 16 h shaking. |
| IP | 0.2 g sediments with 20 mL HCl 1M for 16 h shaking. |
| TP | 0.2 g sediments with 450°C calcination for 3 h; and then 20 mL HCl 3.5 M for 16 h shaking. |
| OP | =TP-IP |
| BAP | =(ex-P)+(Fe/Al-P)+OP |

1. Ruban, V. *et al.* Harmonized protocol and certified reference material for the determination of extractable contents of phosphorus in freshwater sediments - A synthesis of recent works. *Fresen J Anal Chem* **370**, 224-228, doi:DOI 10.1007/s002160100753 (2001).

2. Liu, Q. *et al*. Longitudinal variability of phosphorus fractions in sediments of a canyon reservoir due to cascade dam construction: A case study in Lancang River, China. *Plos One* **8**(12): e83329 (2013). ".

3. Pardo, P. *et al*. Relationships between phosphorus fractionation and major components in sediments using the SMT harmonised extraction procedure. *Analytical And Bioanalytical Chemistry* **376**(2): 248-254 (2003).

**Table S2** Parameters obtained from the modified Langmuir isotherm model*****

| **Site** | **Time** | **ECP_0_ mg/L** | **Q_max_ mg/kg** | **NAP mg/kg** | ***k* L/mg** | ***r^2^*** |
| --- | --- | --- | --- | --- | --- | --- |
| BBF | Apr | 0.311 | 281.7 | 17.03 | 0.203 | 0.979 |
| 4# |  | 0.214 | 658.8 | 16.14 | 0.119 | 0.997 |
| 13# |  | 0.422 | 619.2 | 31.03 | 0.135 | 0.998 |
| 21# |  | 0.137 | 676.9 | 10.86 | 0.138 | 0.998 |
| 30# |  | 0.077 | 1338.5 | 4.72 | 0.065 | 0.999 |
| BBF | Aug | 0.423 | 404.4 | 23.27 | 0.149 | 0.999 |
| 4# |  | 0.160 | 886.3 | 3.56 | 0.049 | 0.999 |
| 13# |  | 0.142 | 1042.4 | 13.81 | 0.093 | 0.999 |
| 21# |  | 0.103 | 1228.7 | 10.54 | 0.083 | 0.999 |
| 30# |  | 0.074 | 1538.9 | 7.18 | 0.063 | 0.999 |
| BBF | Nov | 0.291 | 282.7 | 12.03 | 0.149 | 0.999 |
| 4# |  | 0.244 | 675.9 | 15.81 | 0.100 | 0.999 |
| 13# |  | 0.107 | 1104.5 | 9.91 | 0.087 | 0.999 |
| 21# |  | 0.034 | 1756.6 | 6.53 | 0.064 | 0.999 |
| 30# |  | 0.026 | 1882.0 | 5.98 | 0.061 | 0.999 |
| * *r^2^* is the coefficient of determination of the model; ECP_0_ is the critical concentration below which P would be released from sediments to the water; Q_max_ is the maximum P adsorption capacity in saturation condition; NAP (the Y intercept) refers to the amount of total native (original) adsorbed P; *k* is the bonding energy constant (L/mg). | | | | | | |

**Fig. S1** Average concentrations (mg/L) of different P species and suspended sediments (NTU) in water along the dammed river. The vertical bars indicate the standard errors of the average of three seasons.

1. Corresponding Author: Research Center for Eco-environmental Sciences, Chinese Academy of Sciences, Shuangqing Road 18, Beijing 100085, China.

   *E-mail address*: xyli@rcees.ac.cn (X. Li). [↑](#footnote-ref-1)
